# Intracranial Hemorrhage and Facial Fractures After Nose Blowing and Sternutation: A Case Report

**DOI:** 10.5811/cpcem.1588

**Published:** 2023-08-04

**Authors:** Cameron G. Hanson, Christopher Stewart, Keith Cronovich

**Affiliations:** Henry Ford Health-Macomb, Department of Emergency Medicine, Clinton Township, Michigan

**Keywords:** case report, intracranial hemorrhage, sneezing, blowing nose, orbital fracture

## Abstract

**Introduction:**

Blowing the nose and sneezing are ubiquitous physiologic processes. While exceedingly rare, traumatic injuries have been described. We detail a case of spontaneous intracranial hemorrhage and orbital fractures sustained as a result of these two phenomena in an otherwise healthy adult without known risk factors for bleeding or intracranial hemorrhage.

**Case Report:**

A 79-year-old female presented to the emergency department after blowing her nose with an episode of sneezing following mild epistaxis. She denied any history of trauma, anticoagulation use, bleeding disorders, or pain associated with her symptoms. On examination, she had notable right periorbital swelling. Computed tomography revealed multiple areas of intracranial hemorrhage along with right-sided orbital and zygomatic fractures. After consulting trauma surgery and neurosurgery, we elected to pursue conservative management with repeat imaging. The patient had an uneventful course and was discharged with outpatient follow-up two days later.

**Conclusion:**

To our knowledge, this is the first case described of this constellation of injuries after a relatively benign process. Despite not having increased risk factors for intracranial hemorrhage (anticoagulation use, history of trauma, history of coagulopathy), this patient had severe injuries that presented with few external symptoms. This case serves as a reminder that while physiologic processes are almost always benign, serious traumatic injuries can result. Clinicians should have a low threshold for advanced imaging when there is a high clinical suspicion of facial fractures or more ominous processes.

## INTRODUCTION

Sternutation, or sneezing, is the involuntary, forceful act of expelling irritants from the nasal passages, typically due to noxious stimuli. A universal phenomenon, sternutation is the result of an autonomically triggered event leading to a large pressure build-up in the intrathoracic cavity, followed by release of this pressure into the nasopharyngeal passages at up to 6 kilopascal, or one pound-force per square inch, of pressure with speeds up to 30–40 meters per second (≈70–90 miles per hour).^1^ While this is a common reflex, associated injuries—although rare—have been documented. Injuries are often partially due to the large, pressure build-up effect on upper airway adjacent structures. Traumatic injuries, including orbital blowout fractures, have been reported after sneezing and nose blowing.^2^–^5^ Isolated neurologic cases have been rarely described. We detail a unique case of spontaneous subarachnoid hemorrhage (SAH), subdural hemorrhage, and unilateral orbital blow-out fracture after sternutation in an adult with no prior history of anticoagulation or coagulopathy. To our knowledge, this constellation is the first case of these multiple traumatic injuries after blowing the nose and sternutation.

## CASE REPORT

A 79-year-old female presented to the emergency department (ED) with chief complaint of periorbital edema. The patient stated she had been at a restaurant when she developed spontaneous right-sided epistaxis. Approximately five minutes after bleeding had started, she blew her nose, which led to one episode of sternutation. Following this, her epistaxis had resolved, and she went to the bathroom where she noticed right-sided periorbital edema which prompted her to go to the ED. The patient denied any recent trauma, falls, prior injuries to the head or face, headache, or orbital pain. She had no history of oral anticoagulation use, including aspirin. She denied any history of known osteoporosis, coagulopathy, or bleeding disorders.

Review of systems was remarkable for right eye swelling; however, she denied any sinus pain, periorbital pain, or headaches. On physical examination, there was significant right-sided periorbital swelling without proptosis. There were no signs of periorbital ecchymosis. The patient’s head was atraumatic without abrasions, lacerations, or ecchymosis. Her neurologic exam, including cranial nerve exam testing, was unremarkable. She had notable pain with palpation to the superior orbital rim. There was dried blood from the nares bilaterally, with evidence of a small area of excoriation on the anterior septum on the right, but no active epistaxis. Lab testing, including basic metabolic panel, complete blood count, and coagulation studies, was unremarkable apart from an ethanol level of 100 milligrams/deciliter (mg/dL) (reference range: less than 80 mg/dL). [While we do not believe the patient’s alcohol intoxication played a significant role in her injury, it is a limitation to our report.]

**Image 1:**
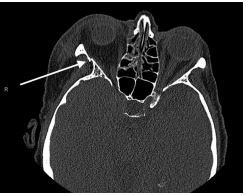
Computed tomography demonstrating right-sided, mildly displaced orbital wall fracture (arrow).

Due to suspicion of orbital injury (including findings of periorbital swelling and bony orbit pain with palpation), the patient underwent computed tomography (CT) of the orbits without contrast. Imaging demonstrated multiple findings including a preseptal hematoma, along with an incomplete right zygomaticomaxillary complex fracture with preservation of the zygomatic arch. A buckle fracture of the lateral wall of the right orbit was also seen, with fractures of the right orbital floor and medial orbital wall without evidence of medial or inferior rectus entrapment [Image 1]. This fracture had propagation through the anterior lateral right maxillary sinus.

**CPC-EM Capsule**

What do we already know about this clinical entity?

*Traumatic injuries very rarely occur after a benign physiologic process such as sneezing.*

What makes this presentation of disease reportable?

*We describe a unique constellation of traumatic injuries, including orbital fractures and intracranial hemorrhage, after the patient blew her nose and sneezed.*

What is the major learning point?

*Advanced imaging should be obtained when there are physical exam findings or symptoms concerning for traumatic injuries*

How might this improve emergency medicine practice?

*By physical exam and clinical suspicion, emergency physicians may identify serious injuries despite a seemingly benign and ubiquitous process.*

**Image 2:**
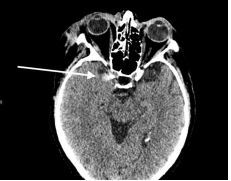
Computer tomography of the head demonstrating foci of subarachnoid hemorrhage (arrow).

**Image 3:**
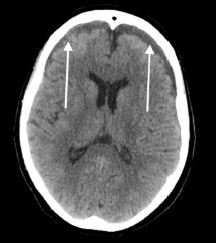
Computed tomography of the head demonstrating right and left subdural hematomas (arrows).

A CT of the head without contrast was also performed, which was remarkable for small foci of SAH over the medial left frontal sulci. There was an additional area of SAH in the sulci of the right temporal parietal junction [Image 2]. Trauma surgery and neurosurgery were consulted and recommended admission with repeat CT in six hours. The following day, the patient’s repeat CT head without contrast showed new bilateral subdural hematomas measuring eight millimeters (mm) on the frontal aspect on the right and seven mm on the left, and SAH in the left parietal lobe [Image 3].

The patient remained stable without any neurologic complaints. Hematology work-up during her hospitalization was unremarkable. Two days following her initial presentation, repeat CT head showed stable findings with no increase in intracranial hemorrhage. Per neurosurgery recommendations, the patient was started on levetiracetam 500 mg twice daily for seven days, and she was subsequently discharged with instructions to follow up with plastic surgery regarding her orbital and facial fractures.

## DISCUSSION

Nose blowing and sternutation is a nearly universal physiologic function that can happen almost daily for a large portion of the population. While rare, traumatic injuries can be associated with this relatively benign process. In 2019 Setzen et al. reviewed 52 case reports of traumatic injuries associated with the act of sneezing.^6^ The authors reported that of these case reports, 65% of patients did not have a prior risk factor for injury (i.e., prior trauma or infection). Of the injuries noted, 25% involved the orbit, including orbital fractures, and 17% involved the nervous system, although SAH and subdural hematoma were not noted in their review.

Our own review of the literature revealed one prior article documenting SAH after sneezing,^7^ and one article describing a spontaneous subdural hematoma after sneezing.^8^ In line with the review by Setzen et al., our review demonstrated that orbital injuries, including orbital fractures and orbital emphysema, were the most common traumatic injuries associated with blowing the nose and sneezing, with the latter being the most described. Neurologic complications were among the rarest injuries and pneumocephalus the most common,^6^,^8^,^9^ but very few articles describe intracranial hemorrhage.

## CONCLUSION

Although the patient had evidence of potential orbital abnormalities prompting further imaging, had this not been present, along with her negative review of systems for associated symptoms (including headache) and lack of increased bleeding risk factors, clinical suspicion for subarachnoid hemorrhage or subdural hematoma may have been low. In this case, physical examination increased the suspicion for possible orbital wall fracture with focal tenderness on palpation around the right orbit, thus prompting a CT of the orbits, which confirmed the diagnosis of complex orbital wall fractures and intracranial blood. Our case demonstrates the extreme end of the spectrum with a constellation of traumatic injuries due to a natural and nearly universal phenomenon. This case report should serve as a reminder that while these physiological processes are ubiquitous and almost always benign, they can cause significant traumatic injuries. Advanced imaging should be obtained when there are physical exam findings or symptoms concerning for traumatic injuries.
